# Early-Life Biological Factors in Functional Neurological Disorder

**DOI:** 10.1192/bjo.2026.11109

**Published:** 2026-06-30

**Authors:** Leonidas Nihoyannopoulos, Bruce Tamilson

**Affiliations:** South West South West London and St. George’s NHS Mental Health Trust, London, United Kingdom

## Abstract

**Aims:**

Functional Neurological Disorder (FND) presents with diverse and often overlappingneurological symptoms, reflecting marked clinical heterogeneity. Research has largely focused on psychological and psychosocial contributors, while biological and developmental health factors earlier in life remain comparatively underexplored. This study examined the prevalence and clinical relevance of early biological factors, neurodevelopmental features, and childhood or adolescent physical health conditions in a cohort of patients with FND.

**Methods:**

We conducted a retrospective analysis of medical records from 282 individuals diagnosed with FND within a tertiary neuropsychiatry service. Extracted data included biological complications occurring around birth, documented neurodevelopmental delays or diagnoses, neurodivergent conditions, and significant medical illnesses during childhood or adolescence. Functional neurological symptoms were grouped into seizure-related, motor, sensory, and cognitive presentations to explore potential associations.

**Results:**

Indicators of early biological and developmental vulnerability were frequently observed. Nearly one-third of patients had recorded perinatal problems, while neurodevelopmental delays and neurodivergent conditions were identified in approximately one-fifth of the cohort. Childhood or adolescent physical illness was common, affecting almost half of the sample.

**Conclusion:**

Early biological, developmental, and physical health factors are commonly documented in individuals with FND and may form part of the broader clinical context in which functional symptoms emerge. Rather than suggesting causation, these findings highlight the importance of considering neurodevelopmental and early-life health vulnerability when conceptualising FND and its varied presentations. Incorporating this perspective may aid in understanding clinical complexity and supporting more individualisedassessment approaches.

